# Assessment of Age and Sex Differences in Risk of 1-Year Mortality After Emergency Department Visits Caused by Alcohol Use

**DOI:** 10.1001/jamanetworkopen.2022.5499

**Published:** 2022-04-04

**Authors:** Daniel T. Myran, Emily Rhodes, Haris Imsirovic, Shannon M. Fernando, Manish M. Sood, Peter Tanuseputro

**Affiliations:** 1 Clinical Epidemiology Program, Ottawa Hospital Research Institute, Ottawa, Ontario, Canada; 2 ICES, Ottawa, Ontario, Canada; 3 Division of Critical Care, Department of Medicine, University of Ottawa, Ottawa, Ontario, Canada

## Abstract

This cross-sectional study assesses age and sex differences in risk of 1-year mortality in patients with emergency department visits due to alcohol compared with the general population in Ontario, Canada.

## Introduction

In the past 2 decades, alcohol-related emergency department (ED) visits have increased in Canada and the US.^1,2^ Previous work has reported that the risk of death is increased among older adults with frequent ED visits related to alcohol.^3^ However, to our knowledge, data are lacking on the clinical importance of ED visits due to alcohol in young adults or individuals with infrequent or singular visits due to alcohol. We evaluated the probability of death in the year after 1 or more alcohol-related ED visits and the differences by age and sex.

## Methods

This repeated cross-sectional study identified all ED visits due to alcohol and deaths from any cause for individuals aged 15 to 59 years in Ontario, Canada, between January 2003 and December 2017 using linked health administrative data through ICES. This study was approved by the privacy and legal offices of ICES and followed the Strengthening the Reporting of Observational Studies in Epidemiology (STROBE) reporting guideline.

We compared the risk of all-cause mortality over 365 days between the general population and individuals with an incident ED visit due to alcohol. Individuals were classified as having a total (including incident visit) of 1, 2, or 3 or more ED visits due to alcohol in the year after the initial visit. Each individual could contribute 1 year of follow-up, which began after their first point of eligibility (eMethods in the Supplement). Poisson models were used to calculate incidence rate ratios (IRRs) with 95% CIs for the risk of death in individuals with ED visits due to alcohol relative to the general population. We ran separate models stratified by age and sex. Data were analyzed from July 2021 through September 2021 using SAS, version 9.4 (SAS Institute).

## Results

A total of 10 197 601 individuals were included (5 119 663 [50.2%] female; mean [SD] age, 36.29 [15.50] years), of which 295 011 individuals (2.9% of all individuals in the study; 184 855 [62.7%] male; mean [SD] age, 32.7 [13.5] years) had 1 or more ED visits due to alcohol (Table). The percent of death within 1 year of 1 or more ED visits due to alcohol (2.0% [5840 of 295 001]) was 4 times greater (IRR, 4.1; 95% CI, 4.0-4.2) than the annual percent of death of individuals in the general population (0.5% [48 574 of 9 902 590]). Older adults, men, and a greater frequency of alcohol-related ED visits were associated with the greatest absolute increases in risk of death. The percent of 1-year mortality was 12.1% (335 of 2774) for men aged 45 to 59 years with 3 or more ED visits due to alcohol and 0.2% (121 of 59 375) for women aged 15 to 29 years with 1 alcohol-related ED visit. Younger age, women, and greater frequency of ED visits due to alcohol were associated with the greatest relative increases in risk of death (Figure).

**Table.** All-Cause Mortality in Year After 1 or More ED Visits Due to Alcohol Compared With General Population by Sex and Age

Description of ED visit	Eligible individuals, No.	All deaths, No. (%)	Death rate per 100 000 person-years, No.	Incidence rate ratio (95% CI)
**Men, age 15-29 y**				
General population^a^	1 605 267	2388 (0.15)	150.1	1 [Reference]
1 ED visit due to alcohol	79 994	264 (0.33)	331.8	2.6 (2.5-2.7)
2 ED visits due to alcohol	3957	34 (0.86)	867.4	5.9 (5.1-6.8)
≥3 ED visits due to alcohol	1066	24 (2.25)	2298.8	12.2 (10.1-14.8)
**Men, age 30-44 y**				
General population^a^	1 818 509	5298 (0.29)	294.5	1 [Reference]
1 ED visit due to alcohol	40 666	638 (1.57)	1592.0	5.2 (5.0-5.4)
2 ED visits due to alcohol	4234	146 (3.45)	3558.0	9.8 (9.1-10.5)
≥3 ED visits due to alcohol	2372	110 (4.64)	4827.4	14.5 (13.4-15.7)
**Men, age 45-59 y**				
General population^a^	1 469 307	21 408 (1.46)	1479.0	1 [Reference]
1 ED visit due to alcohol	44 490	2339 (5.26)	5469.9	3.2 (3.2-3.3)
2 ED visits due to alcohol	5302	504 (9.51)	10 222.2	5.0 (4.8-5.2)
≥3 ED visits due to alcohol	2774	335 (12.08)	13 236.7	6.3 (6.0-6.7)
**Women, age 15-29 y**				
General population^a^	1 671 837	1070 (0.06)	64.5	1 [Reference]
1 ED visit due to alcohol	59 375	121 (0.20)	204.7	3.2 (3.0-3.4)
2 ED visits due to alcohol	2696	11 (0.41)	410.5	10.0 (8.3-12.1)
≥3 ED visits due to alcohol	813	15 (1.85)	1868.1	22.2 (17.5-28.0)
**Women, age 30-44 y**				
General population^a^	1 839 201	3649 (0.20)	199.9	1 [Reference]
1 ED visit due to alcohol	20 616	218 (1.06)	1068.5	6.1 (5.8-6.4)
2 ED visits due to alcohol	2122	43 (2.03)	2059.3	12.7 (11.4-14.2)
≥3 ED visits due to alcohol	1119	45 (4.02)	4146.9	18.2 (16.0-20.6)
**Women, age 45-59 y**				
General population^a^	1 498 469	14761 (0.99)	995.0	1 [Reference]
1 ED visit due to alcohol	20 257	730 (3.60)	3702.5	3.3 (3.2-3.4)
2 ED visits due to alcohol	2141	164 (7.66)	8145.4	6.0 (5.6-6.5)
≥3 ED visits due to alcohol	1017	99 (9.73)	10 441.9	7.1 (6.4-7.9)

Abbreviation: ED, emergency department.

^a^ All individuals in Ontario of same age and sex who did not experience an ED visit due to alcohol during the study period.

**Figure.**
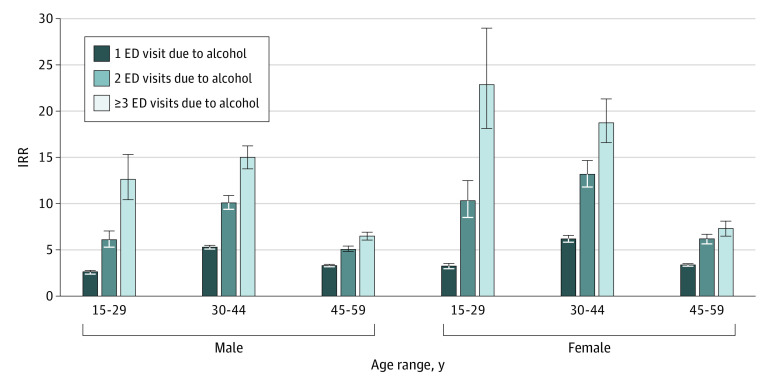
Differences in Rates of Death in Year After Emergency Department (ED) Visits Due to Alcohol Compared With the General Ontario Population Incidence rate ratio (IRR) for rate of death in year after 1 or more ED visits due to alcohol for males and females in 3 age categories compared with the general Ontario population. Error bars represent 95% CIs.

Of the individuals with 1 or more ED visits due to alcohol, 265 398 (90%) had a single ED visit within 1 year. Furthermore, 4310 (73.8%) deaths occurred among individuals with a single alcohol-related ED visit.

## Discussion

The findings of this study suggest a substantial elevation in the risk of mortality for individuals after 1 or more ED visits due to alcohol. The findings also suggest that a single ED visit due to alcohol, even in younger individuals, was associated with a substantial elevation in the risk of death and that most deaths were associated with a single visit.

A study limitation was the inability to identify the role of alcohol in each death. Whereas a single visit may be dismissed as a unique event, and patients may receive minimal follow-up after discharge from the ED, our data suggest that any ED visit due to alcohol is associated with future adverse events. Increases in delivery of interventions may be warranted because ED visits and deaths due to alcohol have been increasing in North America.^1,2,4^ Studies suggest that improving uptake of brief alcohol interventions delivered in the ED and increasing access to medical services for addiction may reduce harm associated with use of alcohol.^5,6^

## Notes

Supplement. **eMethods.** Case Definitions, Data Sources, and Analytical Methods
