# Supplementary material for: Assessment of Age and Sex Differences in Risk of 1-Year Mortality After Emergency Department Visits Caused by Alcohol Use
Source: JAMA Netw Open. 2022 Apr 4;5(4):e225499. doi: 10.1001/jamanetworkopen.2022.5499 (PMC8980906; doi:10.1001/jamanetworkopen.2022.5499)
Supplement: Supplement. — eMethods. Case Definitions, Data Sources, and Analytical Methods [file jamanetwopen-e225499-s001.pdf]

Supplemental Online Content

Myran DT, Rhodes E, Imsirovic H, Fernando SM, Sood MM, Tanuseputro P. Assessment of age and sex differences in risk of 1-year mortality after emergency department visits caused by alcohol use. *JAMA Netw Open*. 2022;5(4):e225499. doi:10.1001/jamanetworkopen.2022.5499

eMethods. Case Definitions, Data Sources, and Analytical Methods

This supplemental material has been provided by the authors to give readers additional information about their work.

© 2022 Myran DT et al. *JAMA Network Open.*

eMethods. Case Definitions, Data Sources, and Analytical Methods

Case Definitions

The primary outcome was all-cause mortality in the year following on or more Emergency Department (ED) visits due to alcohol. ED visits due to alcohol were defined as visits where one of the ICD-10 codes from Table 1 was listed as the main or contributing reason for the ED visit.

Table 1. ICD-10 codes for ED visits due to alcohol

**ICD Code**	**Type of Harm**
F100	Mental and behavioural disorders due to use of alcohol, acute intoxication
F101	Mental and behavioural disorders due to use of alcohol, harmful use
F102	Mental and behavioural disorders due to use of alcohol, dependence syndrome
F103	Mental and behavioural disorders due to use of alcohol, withdrawal state
F104	Mental and behavioural disorders due to use of alcohol, withdrawal state with delirium
F105	Mental and behavioural disorders due to use of alcohol, psychotic disorder
F106	Mental and behavioural disorders due to use of alcohol, amnesic syndrome
F107	Mental and behavioural disorders due to use of alcohol, residual and late-onset psychotic disorder
F108	Mental and behavioural disorders due to use of alcohol, other mental and behavioural disorders
F109	Mental and behavioural disorders due to use of alcohol, unspecified mental and behavioural disorder
E244	Alcohol-induced pseudo-Cushing's syndrome
G312	Degeneration of nervous system due to alcohol
G621	Alcoholic polyneuropathy
G721	Alcoholic myopathy
I426	Alcoholic cardiomyopathy
K292	Alcoholic gastritis
K700	Alcoholic fatty liver
K701	Alcoholic hepatitis
K702	Alcoholic fibrosis and sclerosis of liver
K703	Alcoholic cirrhosis of liver
K704	Alcoholic hepatic failure
K709	Alcoholic liver disease, unspecified
K852	Alcohol-induced acute pancreatitis
K860	Alcohol-induced chronic pancreatitis

© 2022 Myran DT et al. *JAMA Network Open.*

Q99304, Q99305	Maternal care for suspected damage to fetus from alcohol / Fetal Alcohol Syndrome
R780	Finding of alcohol in blood
T510	Toxic effect of alcohol
T519	Toxic effect of alcohol, unspecified
X45	Accidental poisoning by and exposure to alcohol
X65	Intentional self-poisoning by and exposure to alcohol
Y15	Poisoning by and exposure to alcohol, undetermined intent

© 2022 Myran DT et al. *JAMA Network Open.*

Data Sources

Data were obtained using linked health administrative data held by ICES, an independent, non-profit research institute whose legal status under Ontario's health information privacy law allows it to collect and analyze health care and demographic data, without consent, for health system evaluation and improvement. Data on demographic characteristics, ED visits and death were obtained through the following linked, de-identified health administrative databases at ICES: 1) the Registered Persons Database which captures demographic information including age and sex and the date of all deaths; and 2) the National Ambulatory Care Reporting System, which captures all ED visits in Ontario. These datasets were linked using unique encoded identifiers and analyzed at ICES. This project was authorized under section 45 of Ontario's Personal Health Information Protection Act thus not requiring review by a Research Ethics Board.

© 2022 Myran DT et al. *JAMA Network Open.*

Analyses

We present descriptive statistics including rates and proportions for the probability of all-cause mortality in the 365 days following an index event for four groups: the general population of Ontario (excluding those who had one or more ED visit due to alcohol during the study period), and individuals with a total of 1 (i.e., zero additional ED visits due to alcohol), 2 and 3 or more ED visits due to alcohol respectively in the year following the initial ED visit due to alcohol. Each individual in our study could only contribute a maximum of one year to follow up. Individuals were followed until one of the followed occurred a) a year had passed since their first point of eligibility, b) they died or c) they lost coverage for the Ontario Health Insurance Program (the province of Ontario's universal health care coverage). For individuals with ED visits due to alcohol follow up began after their first ED visit due to alcohol in the study period (individuals with more than 1 ED visits due to alcohol in a year were followed for up to 365 days following the final ED visit in that year). For individuals in the general population of Ontario without an ED visit due to alcohol during the entire study period follow up began at the first point of study eligibility.

We used Poisson models offset by the log transformed person time at risk to calculate Incidence Rate Ratios (IRR) with 95% confidence intervals for the risk of death in the three groups of individuals with ED visits due to alcohol (1, 2, 3+ visits in a year) relative to the general population. We ran one overall model and then six separate Poisson models stratified by age and sex (men aged 15-29, 30-44, 45-59 years and women aged 15-29, 30-44, 45-59 years).

© 2022 Myran DT et al. *JAMA Network Open.*
